# Generative artificial intelligence community of practice for research

**DOI:** 10.1111/iwj.14274

**Published:** 2023-06-21

**Authors:** Steve Cohen, Douglas Queen

A recent editorial^1^ discussed the use of generative artificial intelligence (AI) approaches in the management of wounds. As a researcher in the field of wounds, the author provided some insight into how such systems could benefit researchers in this space. The use of such approaches in communities of practice was discussed, providing some of the benefits of this type of approach to researchers. Generative AI can help researchers understand complexity and provide “accurate” summaries of data and outcomes. It can help provide summaries of individual and multiple studies, helping researchers not only understand the content but also provide the outputs to educate others. These communities of practice can also potentially use generative processes to begin drafting manuscripts, this is the automation of the current research processes today. But more study is required to explore the legal and ethical ramifications of the use of generative systems in peer science, as well as training the logic models to provide peer‐validated outcomes.

The editorial mentioned that this was not too far in the future, but do you realise it exists today? Such approaches sum up my field of research for the past few decades, where we worked on the proposed delivery of a personalised information directory for YellowPages Canada (YPG), called YellowSpace. YPG was dropping a 1‐kg phone book on everyone's doorstep. Why not have a personalised digital version of their phonebook for all YellowPages users and businesses across Canada? This approach is more practical and environmentally friendly.

The purpose of YellowSpace was to improve user engagement with the directory listings through behavioural content targeting, providing a unique, personalised view of their national directory for every user across Canada based on their personal preferences and “word of mouth” (family and friends) business logic.

We augmented the directory results by grouping the behavioural actions of close friends and family to make recommendations for food, accommodation, travel, entertainment, and basically anything found in the YellowPages directory. Then, watching the resulting engagement and adjusting the directory recommendations through the user's life cycle. This would dramatically lower search times for relevant listings and increase directory revenue, all while offering a vastly improved user experience with a personalised view of a massive national directory.

From this experience, we have been working on designing and building a software platform to create online communities of practice, where their management is driven by proprietary machine learning/artificial intelligence processes. This approach generatively creates online communities from vast pools of external data and content, providing controlled content surfacing and ML/AI process personalization through analytics, user input, and engagement.

This personalisation provides a unique experience for every member of the community, tailoring what they see with each interaction. Quickly building and populating peer research communities with validated research papers, which are personalised for each viewer's profile, reduces search time and increases engagement.

The community is driven by generative “large language models” (LLM), custom data models, and ML/AI processes to improve the validation and surfacing of appropriate research content, providing an overall personal user experience.

A smart generative feed analyzes the community and its needs and adjusts the content feed to reflect the community profile. Further personalisation can occur through member interaction with content. Based on a community member's profile and area of research focus, the generative process creates clusters of appropriate content and returns results for review; each cluster is presented with a generative abstract. While this personalisation is generative, it can be controlled by the member. Imagine a virtual world that knows your likes and dislikes related to your research and presents only what is of interest to you.

Community members can choose to invoke generative systems to collect additional information about them for inclusion in their profile (BioWindow), including interaction with the community content and members to better profile their likes and dislikes. This also includes within the papers themselves, where community members can roll the cursor over any (hot) paragraph and see a pop‐up list of similar content to that exact paragraph of information, including a generative summary explanation. This focus is designed to narrow down the researcher's train of thought, learn more about the exact area of research they are focused on, and surface more relevant papers, saving them valuable research time.

Such generative artificial intelligence‐controlled communities of practice are the way of the future for researchers.

## References

[1] Queen D . Could wound care benefit from the artificial intelligence storm taking place worldwide. Int Wound J. 2023;20:1337‐1338. 37039743 10.1111/iwj.14171 PMC10088830

